# A Total of 207 Days of Veno-Venous Extracorporeal Membrane Oxygenation Support for Severe COVID-19 Prior to Successful Lung Transplantation: A Case Report

**DOI:** 10.3390/jpm12122028

**Published:** 2022-12-07

**Authors:** Jan Naar, Andreas Kruger, Dagmar Vondrakova, Marek Janotka, Jan Kubele, Robert Lischke, Milena Kolarova, Petr Neuzil, Petr Ostadal

**Affiliations:** 1 Department of Cardiology, Na Homolce Hospital, 15030 Prague, Czech Republic; 2 Department of Clinical Microbiology and ATB Center, Na Homolce Hospital, 15030 Prague, Czech Republic; 3 Prague Lung Transplant Program, 3rd Department of Surgery, Motol University Hospital, 15006 Prague, Czech Republic; 4 Rehabilitation Center, Rehabilitation Hospital of Beroun, 26656 Beroun, Czech Republic

**Keywords:** acute respiratory distress syndrome, coronavirus disease, extracorporeal membrane oxygenation, lung transplant, sepsis

## Abstract

Veno-venous extracorporeal membrane oxygenation (VV-ECMO) is a life-saving treatment for respiratory failure that may serve as a bridge to patient recovery or lung transplantation. In COVID-19, recovery is somewhat unpredictable and occasionally occurs after >100 days on VV-ECMO support. Thus, determining therapy cessation may be difficult. We report the case of a 59-year-old male without specific risk factors admitted to a tertiary center for rapidly progressive respiratory failure due to severe COVID-19, despite aggressive mechanical ventilatory support. Immediate insertion of VV-ECMO was associated with prompt resolution of hypoxemia and hypercapnia; however, all therapeutic efforts to wean the patient from VV-ECMO failed. During the prolonged hospitalization on VV-ECMO, sepsis was the most life-threatening complication. The patient overcame roughly 40 superinfections, predominantly affecting the respiratory tract, and spent 183 days on antimicrobial treatment. Although the function of other organ systems was generally stable, gradually progressive right ventricular dysfunction due to precapillary pulmonary hypertension required increasing doses of inotropes. A successful lung transplantation was performed after 207 days of VV-ECMO support. The present case provides evidence for prolonged VV-ECMO therapy as a bridge to lung transplantation in severe COVID-19 despite numerous, predominantly infectious complications.

## 1. Introduction

Coronavirus disease (COVID-19) currently represents a substantial healthcare burden. The mortality of patients requiring intensive care remains high [1]. Veno-venous extracorporeal membrane oxygenation (VV-ECMO) may be utilized as a life-saving therapy in patients where mechanical ventilation is not able to ensure adequate gas exchange. Compared to non-COVID-19 indications, patients with acute respiratory distress syndrome (ARDS) due to COVID-19 infection require longer ECMO therapy [2,3,4]. The recovery of lung function after >100 days of VV-ECMO support has occasionally been reported in COVID-19 [5]. Rare cases of prolonged VV-ECMO therapy up to 403 days have been published in the pre-COVID-19 era in adults [6,7]. Nevertheless, COVID-19 has unique properties, and we cannot simply extrapolate practice from the long VV-ECMO support of patients with respiratory failure due to other etiologies. Among them, in contrast to non-infectious causes of ARDS, coronavirus-induced immunodeficiency in severe COVID-19 leads to frequent superinfection [8]. We report a case of ARDS caused by COVID-19 with 207 days of VV-ECMO therapy prior to successful lung transplantation.

## 2. Case Report

In February 2021, a 59-year-old Caucasian male with a body mass index of 31 kg/m^2^, no comorbidities, and no chronic medication was admitted to a local hospital, 10 days after the onset of COVID-19 symptoms. Bilateral pneumonia and a positive polymerase chain reaction (PCR) test for severe acute respiratory syndrome *coronavirus 2* (SARS-CoV-2) were revealed upon admission. The sample was not sequenced for subvariant determination; however, the Alpha subtype of SARS-CoV-2 was the most prevalent at that time in the region. The patient was not vaccinated against COVID-19, as the vaccination was only available for older individuals at that time. As the patient was outside the window for remdesivir administration, he received only supportive treatment, including high-flow nasal oxygen therapy and a 10 day course of dexamethasone. Mechanical ventilation was initiated due to progressive respiratory failure after 12 days of hospitalization. After a further 2 days, he was transferred to our center due to gradually progressive hypoxemia and hypercapnia on high ventilatory support with inspiratory pressure of 30–35 cm H_2_O, positive end-expiratory pressure (PEEP) of 6 cm H_2_O, and a fraction of inspired oxygen of 1.0. The ratio of arterial oxygen partial pressure to fraction of inspired oxygen of (PaO_2_/FiO_2_) was 53 mmHg, and the arterial partial pressure of carbon dioxide was 8.4 kPa. VV-ECMO (Cardiohelp, Getinge, Rastatt, Germany) in femoro-jugular configuration was initiated immediately upon arrival. Inflow 25 Fr cannula and outflow 21 Fr cannula were placed into standard position, with the tips reaching the cavoatrial junctions. Anticipating a prolonged need for mechanical ventilation, a percutaneous tracheostomy was performed after another 2 days.

For the first 1.5 months, the patient suffered from frequent hemorrhage, predominantly from a large laceration in the soft palate likely due to nasogastric tube insertion. Repeated sutures, cauterizations, pharyngeal tamponades, and numerous red blood cell transfusion were required. Anticoagulation therapy, initiated upon VV-ECMO implant with unfractionated heparin (UFH) with a target activated partial thromboplastin time (APTT) ratio of 1.5–2.0, needed to be completely withdrawn several times for 1–3 days due to hemorrhage. At that time the APTT ratio dropped to normal or near-normal values without any adverse effect on oxygenator lifespan. In total, 111 blood derivatives were administered to the patient during the 207 day VV-ECMO period, of which 52 were red blood cell transfusions that were used in 40 days. Despite excessive blood derivative consumption, the results of pre-transplantation immunological screening were favorable; the panel reactive antibody test was 3%. After the hemorrhagic complications were resolved, anticoagulation was provided by intravenous enoxaparin, which was administered continuously intravenously maintaining an anti-factor Xa activity of 0.4–0.5. The patient has been predominantly alert since the eighth week on VV-ECMO. Despite the fact that he remained mechanically ventilated via tracheostomy until lung transplantation to enable tracheobronchial toilet due to frequent bacterial superinfections, mechanical ventilation was not an obstacle for daily active rehabilitation. Following that, the patient stopped receiving artificial nutrition and resumed full oral food intake.

Rehabilitation was adjusted according to the physical condition of the patient and was performed twice daily by trained physiotherapists. It consisted of passive and active physiotherapy using proprioceptive neuromuscular facilitation techniques and breathing exercises. Training on a MOTOmed stationary bicycle (RECK-Technik, Betzenweiler, Germany), also enabling passive rehabilitation, was added. When in good condition, the patient was able to cycle actively with a lower level of resistance for 15–20 min twice daily, achieving 2 km distance each training period. Cycling in a semi-recumbent position was feasible despite an outflow cannula in the femoral vein. Physiotherapy was accompanied by ergotherapy to practice fine motor skills and self-management.

**Figure 1:**
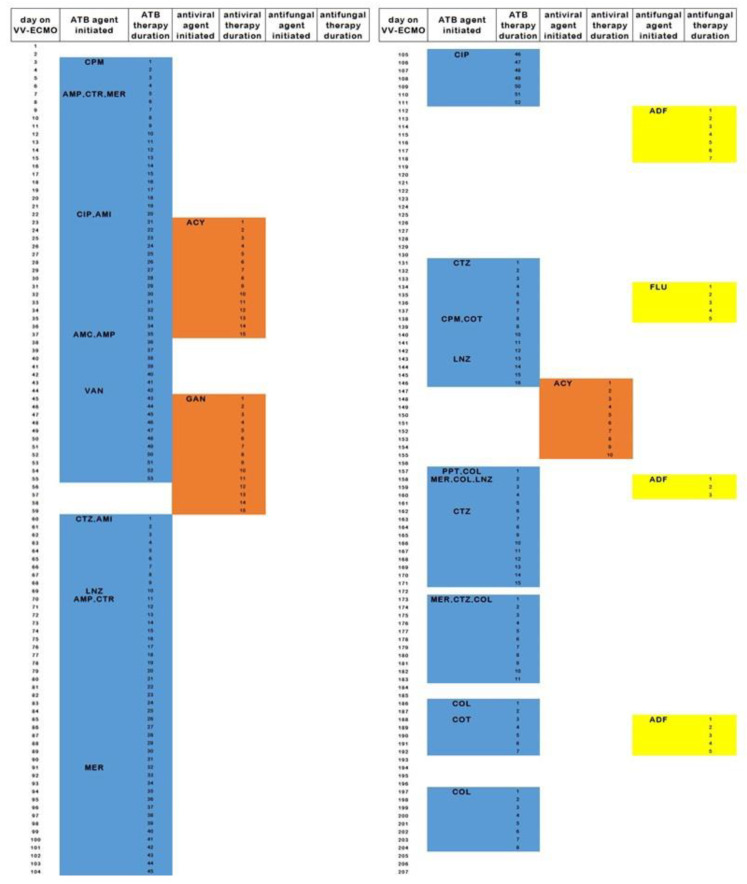
Type and duration of antimicrobial therapy during 207 days of VV-ECMO support until lung transplantation. Antibiotic therapy is in blue, antiviral therapy in orange and antifungal therapy in yellow. ACY = aciclovir, ADF = anidulafungin, AMC = amoxicillin + clavulanic acid, AMP = ampicillin, ATB = antibiotic, CIP = ciprofloxacin, COL = colistin, COT = co-trimoxazole, CPM = cefepime, CTR = ceftriaxone, CTZ = ceftazidime, FLU = fluconazole, GAN = ganciclovir, LNZ = linezolide, MER = meropenem, PPT = piperacillin/tazobactam, VAN = vancomycin.

Active rehabilitation was, however, frequently interrupted by severe infections. The predominant origin of bacterial superinfections was the respiratory tract. Five bloodstream infections and three reactivations of herpetic viruses were documented. One ECMO cannula replacement was performed after 70 days of VV-ECMO support due to enterococcus bloodstream infection (ipsilateral internal jugular vein and contralateral femoral vein accesses were used). The patient was treated with an antimicrobial agent for 183 days in the 207 day pre-transplant VV-ECMO period. That agreed with the 662 defined daily doses of antibiotic, antiviral, and antifungal medication. Antimicrobial therapy details are shown in Table 1 and Table 2 and Figure 1.

For the first four months, the patient continuously received systemic corticosteroid therapy with methylprednisolone (10–40 mg per day) to positively influence the pulmonary recovery process. The patient was turned to a prone position four times for 8–16 h period. Due to the lack of any clear signs of improvement and the development of facial pressure ulcers, we abandoned prone position management. Pulmonary involvement was extensive, according to X-rays and computed tomography scans. A protective ventilation regime using inspiratory pressures of 14–16 cm H_2_O and PEEP of 10–12 cm H_2_O was applied most of the time. Tidal volume and lung compliance remained depressed: maximal tidal volumes reached 300–350 mL with strong patient effort but were only around 100 mL during sedation on mechanical ventilation using peak pressures up to 30 cm H_2_O. Static compliance was 8–20 mL/cm H_2_O, accordingly. As the patient remained fully dependent on extracorporeal membrane oxygenation, he was accepted as an urgent candidate for lung transplantation at day 90 of VV-ECMO therapy. At the time of lung transplant acceptance, VV-ECMO parameters were: blood flow of 4.4 L/min, gas flow of 5.5 L/min, and oxygen fraction of 0.9. The oxygenator was exchanged 10 times, with the average lifespan of one oxygenator being 19 days.

After 10 weeks of VV-ECMO support, severe precapillary pulmonary hypertension developed, leading to right ventricular dilation and systolic dysfunction despite sildenafil therapy, requiring continuous administration of inotropes (dobutamine and milrinone). According to transthoracic echocardiography, the estimated systolic pulmonary pressure was 70 mmHg, the right ventricular basal diameter from the apical four chamber view was 47 mm, and the tricuspid annular plane systolic excursion (TAPSE) was around 17 mm.

After 207 days on VV-ECMO, the patient underwent successful bilateral lung transplantation. Cold ischemia in the right lung was 117 min and 217 min in the left lung. The surgery lasted almost 7 h and perioperatively it was necessary to switch to veno-arterial (VA) ECMO support. The ECMO system was removed after 212 days of extracorporeal therapy and 236 days from the onset of COVID-19 symptoms.

The postoperative status was favorable, with good graft function. Later, rehabilitation was impaired by bilateral femoral neck fractures due to severe osteoporosis, likely due to prolonged corticosteroid therapy and immobilization. Long-term low-molecular-weight heparin therapy may have also contributed to decreased bone mineral density [9]. The patient died after 6 months of lung transplantation and 407 days in the hospital due to sepsis originating from the respiratory tract.

## 3. Discussion

The present report describes a case of extremely long VV-ECMO support in severe respiratory failure due to COVID-19 that bridged the patient to successful lung transplantation. The hospitalization was complicated by numerous infections and progressive right heart failure. To our knowledge, this is the longest published VV-ECMO support in COVID-19 worldwide.

VV-ECMO treatment of COVID-19 ARDS has different features compared to other types of respiratory failure. In-hospital mortality in COVID-19 patients with ARDS requiring VV-ECMO support reaches 50% [4]. In the late pre-COVID-19 era, the mean time on VV-ECMO was around 11 days, whereas it is 26 days in COVID-19 ARDS patients [2,3]. The longest reported VV-ECMO support in an adult patient was 403 days in a 50-year-old male with idiopathic pulmonary fibrosis [7]. It seems that the short-term outcome of patients requiring lung transplantation due to COVID-19 is comparable with the individuals undergoing transplant surgery due to non-COVID etiologies [10].

Prolonged VV-ECMO therapy in COVID-19 patients presents several challenges, some of which are general and related to the long duration of VV-ECMO support. Outflow cannula dislocation, fortunately prevented in our case, represents a nightmarish complication with fatal consequences. Regular cannula fixation checks are required, especially in awake patients undergoing intensive active rehabilitation. Decreased staff alertness after hundreds of days of hospitalization is expected but must be avoided. There are also extreme demands on nursing staff to prevent pressure ulcers and on physiotherapists to ensure optimal musculoskeletal status before potential transplant surgery. Caring for a conscious, immobilized patient with an uncertain prognosis and a communication barrier (mechanical ventilation with limited phonation) brings enormous stress that affects the patient as well as all caregivers, especially nursing and medical staff. Continuous positive motivation of the patient along with pharmacological control of depression and anxiety was essential in the present case.

There are some issues specific to COVID-19. These patients more often suffer from bacterial, viral, and fungal superinfections due to apparent immunodeficiency, especially after a severe SARS-CoV-2 infection with devastating lung damage [8]. Opportunistic infections occur frequently and must be searched for proactively [11]. Despite the fact that the causes and mechanisms of superinfections in COVID-19 may be multifactorial [12], SARS-CoV-2 virus-induced impairment of cellular immunity may play an important role in severe COVID-19 infection [13]. Close cooperation with infectious disease specialists, microbiologists, and clinical pharmacists is essential to treat coinfections efficiently and promptly and to avoid antibiotic toxicity and resistance. A total of 109 microbiologist consultations and 40 antimicrobial therapy initiations were registered during the 207 days on VV-ECMO before transplantation. Finding a proper organ donor may be more difficult, as COVID-19 recipients need relatively smaller grafts due to restriction of the pleural cavity caused by fibrotic processes accompanying severe pulmonary destruction, resulting in a longer time on the transplant waiting list.

Another important issue is the need for and frequency of ECMO cannula exchanges. In the above-mentioned case of idiopathic pulmonary fibrosis, ECMO therapy was withdrawn due to a lack of venous access sites after 10 ECMO cannula replacements [7]. The optimal strategy is highly debatable. Apart from the depletion of insertion sites, a preventative strategy with frequent cannula exchange brings a considerable risk of hypoxic cardiac arrest in fully oxygenator-dependent patients and a potentially lethal venous mechanical complication during fast cannulation. We exchanged ECMO cannulas just once for an enterococcal bloodstream infection. Nevertheless, it must be stated that in the event of an aggressive bloodstream infection such as *Staphylococcus aureus* or *Candida* spp., we would consider and recommend another exchange. Therefore, the frequency of ECMO cannula exchange must be strictly individualized. After thorough consideration of risks versus benefit in the fully oxygenator-dependent patient, we did not attempt a single-site cannulation strategy using a double-lumen jugular cannula. In retrospect, knowing that prolonged immobilization markedly contributed to osteoporosis and pathological fractures despite regular rehabilitation, including stationary cycling, we would seriously consider single-site jugular cannulation.

Regarding the amount of red blood cell substitution we admit, that in light of recent evidence concerning the target hemoglobin level of VV-ECMO patients, we would be more conservative in administering transfusions to avoid the risk of blood derivative-induced immunomodulation [14].

Progressive right heart failure is a common complication of pulmonary failure of any origin treated by prolonged VV-ECMO support [7,15,16]. In the present case, apart from frequent septic events, advanced right ventricular dysfunction became the most threatening complication and an important negative prognostic factor requiring consideration at the time when the final decision regarding lung transplantation was made. Fortunately, pulmonary hypertension and right ventricular dysfunction proved to be fully reversible in the present case.

## 4. Conclusions

The present case report provides evidence that prolonged VV-ECMO use in ARDS due to COVID-19 may serve as a bridge to lung transplantation, despite numerous complications and therapeutic difficulties. A multidisciplinary approach, including microbiology, infectious disease, and pharmacology specialists, and full engagement of the medical team is essential. The risk of subsequent development of severe osteoporosis must be considered with excessively long VV-ECMO support.

## Figures and Tables

**Table 1:** Details of antimicrobial therapy during 207 days of VV-ECMO support until lung transplantation.

Materials for cultivation (*n*)	499
Blood cultures (*n*)	97
Positive blood cultures (*n*)	22
Blood volume taken for blood cultures (mL)	970
Cultivated catheters (*n*)	31
Lower respiratory tract materials (*n*)	106
Validated bloodstream infections (*n*)	5
Bloodstream infection agents	*Enterococcus faecalis*, *Klebsiella aerogenes*, *Enterococcus faecium*, and *Staphylococcus saprophyticus*.
Microbiologist consultations (*n*)	109
Antibiotic therapies >24 h except prophylactic (*n*)	33
Antiviral therapies (*n*)	3
Antifungal therapies (*n*)	4
Defined daily dose of all antimicrobial drugs (days)	632
Defined daily dose of antibiotics (days)	564
Total dose of antibiotics (g)	2135

**Table 2:** Antimicrobial doses during 207 days of VV-ECMO support until lung transplantation.

Antimicrobial Agent	Type	Defined Daily Dose (Days)	Therapy Duration (Days)	Total Dose (g)
Aciclovir	AV	9	24	36
Amikacin	ATB	12	8	12
Amoxicillin/clavulanic acid	ATB	12	9	43.2
Ampicillin	ATB	162	66	972
Anidulafungin	AF	21	17	2.1
Cefepime	ATB	19.5	13	78
Ceftazidime	ATB	69	36	276
Ceftriaxone	ATB	112	56	224
Ciprofloxacin	ATB	33	22	26.4
Colistin	ATB	14.7	38	132.3
Co-trimoxazole	ATB	8	8	15.4
Fluconazole	AF	8	4	0.8
Ganciclovir	AV	30	15	15
Linezolide	ATB	8.5	8.5	10.2
Meropenem	ATB	94.7	48	284.1
Piperacillin/tazobactam	ATB	2	1	28
Vancomycin	ATB	16.5	11	33

AF = antifungal, ATB = antibiotic, AV = antiviral.

## Data Availability

More detailed data regarding antimicrobial therapy in the present case are available from the corresponding author upon reasonable request.
